# Physiological and lipidomic response of exogenous choline chloride alleviating salt stress injury in Kentucky bluegrass (*Poa pratensis*)

**DOI:** 10.3389/fpls.2023.1269286

**Published:** 2023-08-31

**Authors:** Zhi-Fang Zuo, Yan Li, Xin-Feng Mi, Yong-Long Li, Chen-Yuan Zhai, Guo-Feng Yang, Zeng-Yu Wang, Kun Zhang

**Affiliations:** College of Grassland Science, Qingdao Agricultural University, Qingdao, Shandong, China

**Keywords:** choline chloride, salt stress, phospholipids, glycolipids, turfgrass

## Abstract

**Introduction:**

Choline participates in plant stress tolerance through glycine betaine (GB) and phospholipid metabolism. As a salt-sensitive turfgrass species, Kentucky bluegrass (*Poa pratensis*) is the main turfgrass species in cool-season areas.

**Methods:**

To improve salinity tolerance and investigate the effects of choline on the physiological and lipidomic responses of turfgrass plants under salinity stress conditions, exogenous choline chloride was applied to Kentucky bluegrass exposed to salt stress.

**Results:**

From physiological indicators, exogenous choline chloride could alleviate salt stress injury in Kentucky bluegrass. Lipid analysis showed that exogenous choline chloride under salt-stress conditions remodeled the content of phospholipids, glycolipids, and lysophospholipids. Monogalactosyl diacylglycerol, digalactosyl diacylglycerol, phosphatidylcholine, phosphatidylethanolamine, phosphatidylserine, and lysophosphatidylcholine content were increased and phosphatidic acid content were decreased in plants after exogenous choline chloride under salt treatment. Plant leaf choline content increased, but GB was not detected in exogenous choline chloride treatment plants under nonstress or salt-stress conditions.

**Discussion:**

GB synthesis pathway related genes showed no clear change to choline chloride treatment, whereas cytidyldiphosphate‐choline (CDP‐choline) pathway genes were upregulated by choline chloride treatment. These results reveal that lipid remodeling through choline metabolism plays an important role in the salt tolerance mechanism of Kentucky bluegrass. Furthermore, the lipids selected in this study could serve as biomarkers for further improvement of salt-sensitive grass species.

## 1 Introduction

Salt stress, caused by soil salinization, is an important factor for plant distribution worldwide and greatly affects plant development and productivity (Deinlein et al., 2014). Salt stress primarily causes osmotic and ionic toxicity in plant cells, which induces secondary effects of oxidative stress that greatly damage cellular components, such as membrane lipids (Zhu, 2016). Halophytes and salt-tolerant glycophyte cultivars are tolerant to saline conditions and exhibit various mechanisms, including complex physiological traits, molecular or gene networks, and metabolic pathways (Annunziata et al., 2019). The research about plant salinity stress response, such as through osmotic adjustment (OA), which maintains cell membrane integrity and stability, could provide a combination of molecular tools for developing salt-tolerant varieties in saline environments. Therefore, it is an effective method to elevate plant salinity tolerance through increasing membrane stability (Lv et al., 2021).

Salinity enhances lipid peroxidation, which, in turn, impairs cellular membrane permeability. Phospholipids and glycolipids are two basic lipids in plant cell membranes and function as maintaining membrane structure and regulating plant salt stress tolerance (Mansour, 2013; Chalbi et al., 2015). Phospholipids, like phosphatidic acid (PA), phosphatidylcholine (PC), phosphatidylethanolamine (PE), could work as signaling compounds in plants exposed to abiotic stress (Hou et al., 2016; Han and Yang, 2021). In structural phospholipids, PC and PE occupy a large proportion and the remainder consists of phosphatidylglycerol (PG), phosphoinositides (PI), phosphatidylserine (PS), and PA (Furt et al., 2011). The minor lipids PA and phosphatidylinositol bisphosphate (PIP2) act as salt stress-signaling lipids that rapidly accumulate in rice (*Oryza sativa*) leaves, as detected using ^32^P labeling (Darwish et al., 2009). Platre et al. (2018) showed that PS and PA are required to generate the electrostatic signature of the plant plasma membrane, so their function could be separated based on their varying surface charges. PC is induced by salt stress and might function in the maintenance of membrane structure and function. Liu et al. (2015) reported that PE might be indirectly converted to PC in *Arabidopsis via* a hypothetical methylation pathway involving the synthesis of lysophosphatidylethanolamine (LysoPE), its methylation to lysophosphatidylcholine (LysoPC), and conversion to PC. PC could be synthesized by choline from the cytidyldiphosphate‐choline (CDP‐choline) pathway (Annunziata et al., 2019). In maize roots, PC levels decreased significantly with the increased expression of some phospholipase genes under saline-alkaline stress, suggesting there was a PC mediated lipid reprogramming under stress condition (Xu et al., 2021).

Glycolipids, like monogalactosyl diacylglycerol (MGDG) and digalactosyl diacylglycerol (DGDG), are major chloroplast thylakoid membranes components and primarily affect the photosystem II (PSII) characteristic (Mizusawa and Wada, 2012). In addition, a higher ratio of DGDGs/MGDGs to PCs/PEs is an indicator of the maintenance of membrane fluidity in response to abiotic stress in different plant species. It has been reported that the DGDG/MGDG ratio is response to salt stress (Guo et al., 2019). In transgenic tobacco (*Nicotiana tabacum*) with *OsMGD* gene, plants had a high salt tolerance, increased MGD activity and higher DGDG/MGDG ratios, which revealed a more stable thylakoid membrane in transgenic plant (Wang et al., 2014). In addition, the variation of membrane lipids compositions and fatty acid desaturation under salt stress condition have been identified as a common strategy for plant *via* affecting membranes mobility and stress signal transduction. (Magdy et al., 1993; Mansour, 2013).

Glycine betaine (GB) could synthesized from choline with two key enzyme, choline monooxygenase (CMO) and betaine aldehyde dehydrogenase (BADH) (Annunziata et al., 2019). In halophyte *Chenopodium quinoa*, choline accumulation could help to increase GB to improve salt tolerance (Pottosin et al., 2014). For some species like kidney bean (*Phaseolus vulgaris*) and wheat (*Triticum aestivum*), GB is a highly abundant compatible solute during the regulation of osmotic stress (Kreslavski et al., 2001; Salama et al., 2011). In *seashore paspalum*, the application of choline induced salt tolerance both in different salt sensitivity cultivars with remodeling the lipid profile and increase the GB content (Gao et al., 2020). However, not all plant species has high levels of GB, such as *Arabidopsis thaliana* and some grass species (Sakamoto and Murata, 2002; Hu et al., 2012). The exogenous treatment of choline or GB has effectively enhanced plant tolerance to abiotic stress, particularly for these low GB level species. In the C3 *Poa* turfgrass, foliar application of GB alleviated the physiological injury caused by drought or salt stress by maintaining membrane stability and active SOD, POD, and APX activity (Yang et al., 2012). In rice, choline priming could mitigate the salt stress during seed germination (Huang et al., 2023). Therefore, other metabolic pathways, such as the phospholipid and glycolipid reprograming through CDP-choline pathway, might play important role in dealing with salt stress in these species.

In this study, we examined the physiological response of Kentucky bluegrass with exogenous choline chloride under salt stress. Characterizing the GB and lipids (mainly phospholipids and glycolipids) metabolism that may be involved in the choline regulation of salt tolerance could provide theoretical basis for the important role of lipid metabolism in CDP-choline pathway during plant under salt stress condition. The selected lipid species in our study could be molecular markers for further salt stress tolerance improvement in salt-sensitive grass species.

## 2 Materials and methods

### 2.1 Plant material and treatment

Kentucky bluegrass (‘Diva’) seeds were sown in plastic pots (length 10 cm, width 7 cm, height 8.5 cm) filled with fritted clay. After 2-month pre-cultivation, uniform-size seeding were transferred to hydroponic conditions (0.5×Hoagland’s nutrient solution) in a growth chamber. Seedlings were cultivated for 21 days to adapt to the environment before exposure to the experimental treatments.

Plants were foliar-sprayed with 1 mM choline chloride until it dripped from the plants based on the previous study (Gao et al., 2020). Choline chloride treatment was performed 7 days prior to the start of stress application and continued at 7-day intervals during the experimental processing. 100 mM sodium chloride (NaCl) was added for the salt stress treatment. The salt stress was starting with 30 mM NaCl, and then gradually increased to 100 mM in two days. Each treatment had four replicates. Physiological measurements were performed 7, 14, and 21 days after treatment with 100 mM NaCl. Leaf was sampled at 14 days after salt stress treatment, then washed with deionized water and stored in -80°C for GB and choline content measurements, gene analysis, and lipid analysis. The plant growth state was photographed after 21 days of salt stress treatment.

### 2.2 Physiological measurements

The leaf Fv/Fm, electrolyte leakage, relative water content, and chlorophyll content was measured according to the previous study (Zhang et al., 2019; Zhang et al., 2020). The leaf osmotic adjustment was determined following a previous study (Gao et al., 2020). Quantification of GB and choline was performed using HPLC–MS based on a method described previously with modifications (Koc et al., 2002).

### 2.3 Lipid extraction

Lipid extraction was performed according to previous studies (Zhang et al., 2019). 1 mL lipid extract (methyl alcohol:methylene dichloride=1:1) was added and vortexed for 30 min. Added 300 μL ultra-pure water and vortexed for 1 minute and incubated at 4°C for 10 minutes. After centrifugation at 12000 rpm and 4°C for 3 minutes, 400 μL supernatant evaporated with nitrogen gas and concentrated to dry. Then, the sample was redissolved in 200 μL isopropanol. After filtered with filter membrane, the solution was ready for LC–MS/MS analysis.

### 2.4 HPLC and ESI–MS/MS conditions

The lipid extracts were analyzed with an LC–ESI- QTRAP/MS system (UPLC, Exion LC AD; MS, QTRAP 6500+ System). The ESI source operation parameters were as follows: ion source, turbo spray; source temperature 500°C; ion spray voltage 5500 V(Positive), −4500 V (Negative); Ion source gas 1, gas 2, and curtain gas were set at 45, 55, and 35 psi, respectively. The lipid unsaturation index was calculated as previously described (Su et al., 2009).

### 2.5 qRT-PCR analysis

Leaf RNA was isolated with the RNA EASY Fast Kit (TIANGEN, China). cDNA synthesis and qRT-PCR analyses was performed with FastKing One Step RT-PCR Kit (TIANGEN, China) on QuantStudio 1 Plus (Applied biosystems, USA). The qRT-PCR primers are listed in **Supplementary Table S1**. The *PpGADPH* gene was used as a reference control for qRT-PCR analysis according to a previous study (Niu et al., 2017). The gene expression was calculated using the 2^-△△Ct^ method (Livak and Schmittgen, 2001). Each qRT-PCR analysis was performed in triplicate.

### 2.6 Statistical analysis

The data were analyzed with Spss 17.0 (SPSS Inc., Chicago, IL, USA). Fisher’s protected least significant difference (LSD) test was performed to calculate the significant differences (*P* < 0.05).

## 3 Results

### 3.1 The growth and physiological changes of Kentucky bluegrass

**Figure 1:**
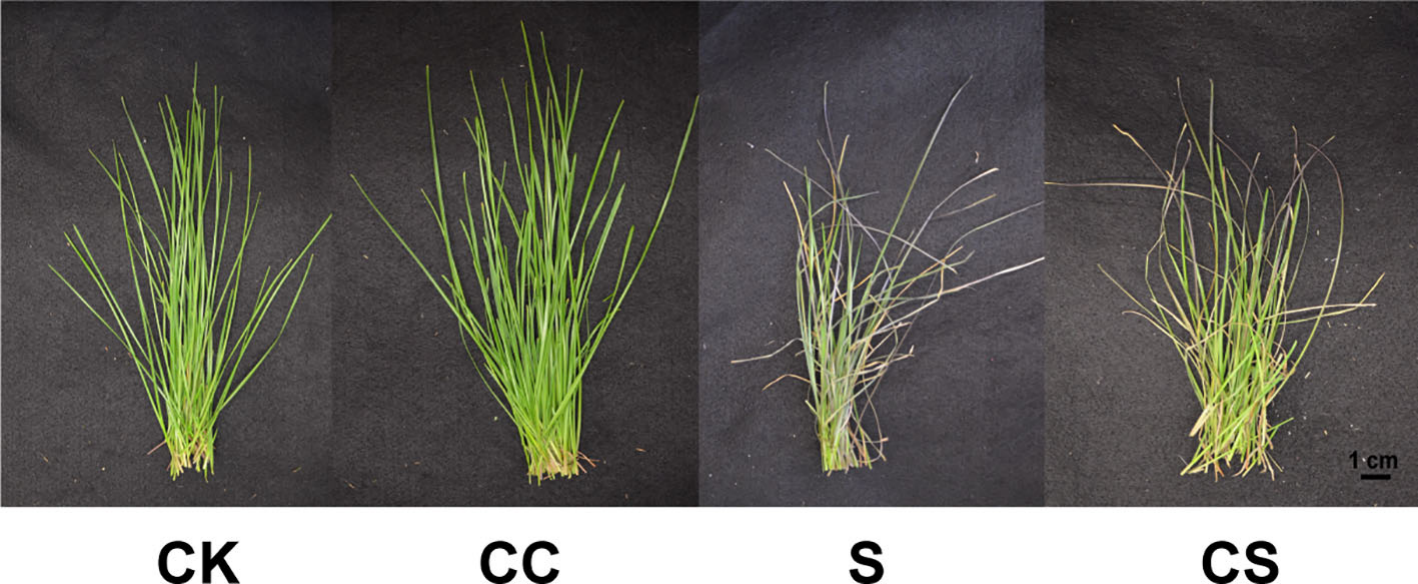
Growth of Kentucky bluegrass treated with choline chloride and salt stress. The photographs were taken after 21 days of treatment. CK, control treatment; CC, choline chloride treatment; S, salt treatment; CS, choline chloride and salt treatment, the same as below.

Kentucky bluegrass plants were cultured in nutrient solution were foliar-sprayed with choline chloride and subjected to a salt solution for 21 days (**Figure 1**). Choline chloride treatment had improved the plant growth with more leaves compared to untreated plant. Under salt stress condition, greener and fewer wilted leaves were observed in the choline chloride treatment plants than untreated plant. Growth characteristics showed that exogenous choline chloride increased the salt tolerance of Kentucky bluegrass.

**Figure 2:**
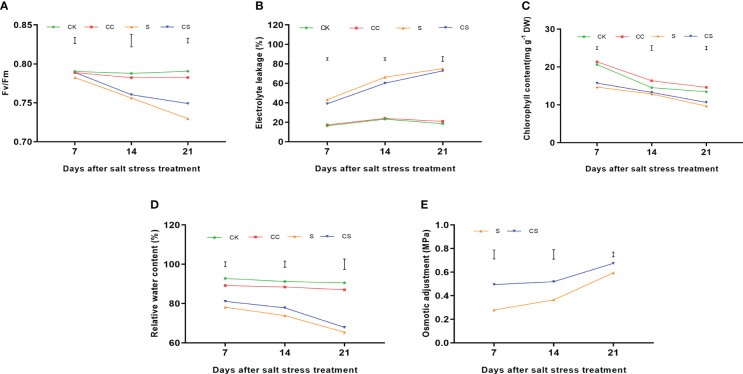
The physiological indicators of Kentucky bluegrass with choline chloride and salt stress treatments. **(A)** Fv/Fm. **(B)** Electrolyte leakage. **(C)** Chlorophyll content. **(D)** Relative water content. **(E)** Osmotic adjustment. All data are means ± SE for three biological replicates. The vertical bars indicate the values of LSD at *P*-value=0.05.

In addition, physiological indicators were determined to illustrate the mechanism that increased salt tolerance in Kentucky bluegrass after the exogenous application of choline chloride. The Fv/Fm ratio decreased after salt stress treatments (**Figure 2A**). After 7 days of treatment, the difference of Fv/Fm ratio among the four treatments was not significant. After 14 days of treatment, the Fv/Fm ratio was significantly declined under stress conditions compared to the nonstress condition, whereas choline chloride did not affect the Fv/Fm ratio under stress and nonstress conditions. At 21 days of treatment, plants had a significantly higher Fv/Fm ratio in choline chloride treated plants, whereas salt treatment significantly decreased the Fv/Fm ratio compared to the nonstress plants. Electrolyte leakage were upregulated in both choline chloride untreated and untreated plants after salt stress (**Figure 2B**). At 7 and 14 days after salt stress, exogenous choline chloride resulted in lower EL (9.62% and 9.37%, respectively) than that of untreated plants. Chlorophyll content in all treatment were decreased during salt stress (**Figure 2C**) and it was significantly higher in choline chloride treated plants than that in untreated plants 7 (7.03%) and 21 days (10.00%) after salt treatment, whereas exogenous choline chloride significantly increased the chlorophyll content 14 and 21 days after the non-stress treatment. The relative water content in all treatment were decreased during salt stress (**Figure 2D**). Plants had significantly higher relative water content in choline chloride treated plants than that in untreated plants 7 (3.86%) and 14 days (5.37%) after salt treatment. Salt treatment significantly declined the relative water content compared to the nonstress treatment during days 7–21 of salt stress (**Figure 2E**). In addition, OA was detected in the leaves of the four treatments, and this value increased during the salt stress treatment. Significantly higher OA was found in choline chloride-treated plants from 7 to 21 days of stress treatment, with increases of 77.61%, 42.54%, and 13.55%, respectively, than in untreated plants.

### 3.2 The leaf lipidomic changes of Kentucky bluegrass

After 14 days of salt stress, the leaf tissues of Kentucky bluegrass were collected for lipidomic analysis. The leaf lipidomic change profiles are shown in **Figure S1**. In total, 150 lipid molecular species were tested, including DGDG, MGDG, PA, PC, PE, PG, PI, PS, and LysoPC in Kentucky bluegrass treated with choline chloride and salt stress.

**Figure 3:**
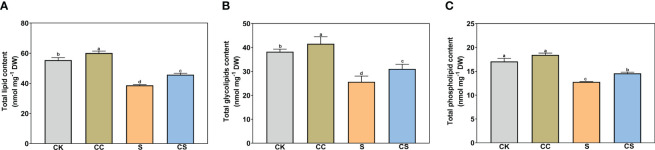
The contents of total lipid, glycolipid, and phospholipid of Kentucky bluegrass with choline chloride and salt stress treatments. **(A)** Total lipid content. **(B)** Total glycolipid content. **(C)** Total phospholipid content. All data are means ± SE for three biological replicates. Different letter indicated the significance at *P* < 0.05, the same as below.

As shown in **Figure 3**, the total lipid, total glycolipid, and total phospholipid contents significantly decreased after salt stress treatment. The total lipid content increased by 8.43% and 18.41% with exogenous choline chloride under nonstress and salt stress treatment (**Figure 3A**). The content of total glycolipid was significantly higher in choline chloride -treated plants than that in untreated nonstress control (8.67%) and salt-stressed plants (21.03%) (**Figure 3B**). For total phospholipids, an increase was found only in choline chloride-treated plants compared to non-treated plants under salt stress conditions (**Figure 3C**).

**Figure 4:**
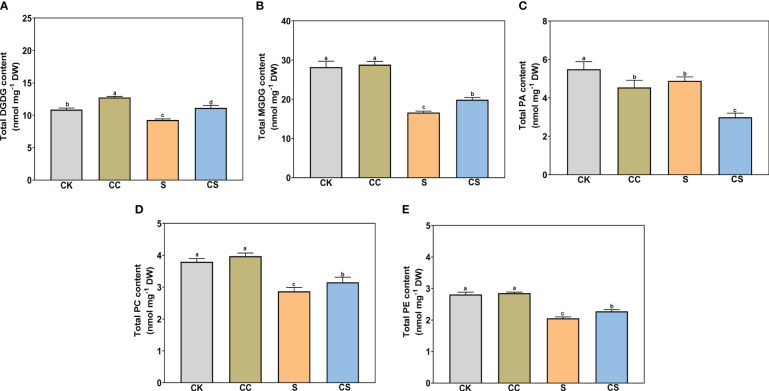
The contents of total DGDG, MGDG, PA, PC, and PE of Kentucky bluegrass with choline chloride and salt stress treatments. **(A)** Total DGDG content. **(B)** Total MGDG content. **(C)** Total PA content. **(D)** Total PC content. **(E)** Total PE content. All data are means ± SE for three biological replicates. Different letter indicated the significance at P < 0.05.

**Figure 5:**
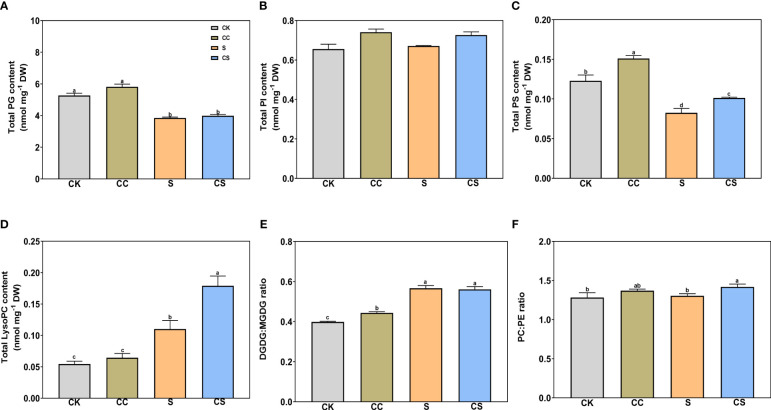
The contents of total PG, PI, PS, and LysoPC, and the ratio of DGDG:MGDG and PC:PE of Kentucky bluegrass with choline chloride and salt stress treatments. **(A)** Total PG content. **(B)** Total PI content. **(C)** Total PS content. **(D)** Total LysoPC content. **(E)** DGDG:MGDG ratio. All data are means ± SE for three biological replicates. Different letter indicated the significance at P < 0.05.

In glycolipids, the total DGDG and MGDG contents were both significantly declined with or without choline chloride treatment under salt stress (**Figures 4A, B**). Exogenous choline chloride significantly increased the total DGDG content under nonstress and salt-stress conditions by 17.37% and 20.15%, respectively, and increased the total MGDG content by 19.76% under salt-stress conditions compared to the control. In phospholipids, the total PA, PC, and PE (**Figures 4C–E**) and total PG and PS (**Figures 5A, C**) contents significantly decreased after salt stress treatment with or without choline chloride treatment. Exogenous choline chloride significantly increased the total PC content by 9.73% under salt-stress conditions, decreased the total PA content by 17.33% and 38.70% under nonstress and salt-stress conditions, respectively, and increased the total PS content by 22.76% and 22.73% under nonstress and salt-stress conditions, respectively. The PI content had no significant changes under different treatments (**Figure 5B**). The lysophospholipid (LysoPC) content also had no significant difference with choline chloride treatment under nonstress conditions, but the difference was significant under salt-stress conditions (S, 0.11 nmol mg^-1^, CS 0.18 nmol mg^-1^) (**Figure 5D**). In addition, exogenous choline chloride significantly increased the DGDG:MGDG ratio compared with that in treated plants under nonstress conditions (**Figure 5E**). Under salt treatment, the PC:PE ratio was significantly higher (8.68%) in choline chloride -treated plants than in untreated plants (**Figure 5F**).

### 3.3 The variation of different lipid molecular species in Kentucky bluegrass

**Figure 6:**
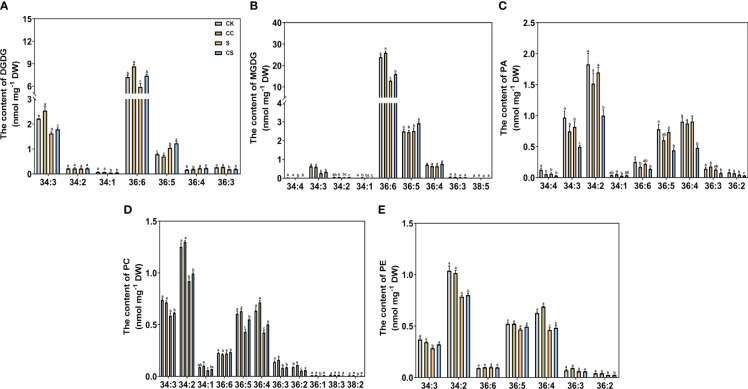
The specific molecular species of DGDG, MGDG, PA, PC, and PE of Kentucky bluegrass with choline chloride and salt stress treatments. **(A)** The content of DGDG content. **(B)** The content of MGDG content. **(C)** The content of PA content. **(D)** The content of PC content. **(E)** The content of PE content. All data are means ± SE for three biological replicates. Different letter indicated the significance at P < 0.05.

**Figure 7:**
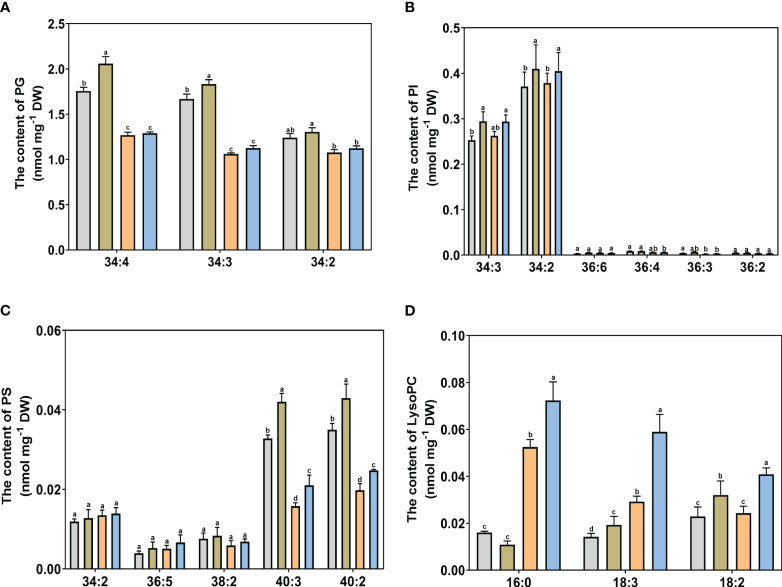
The specific molecular species of PG, PI, PS, and LysoPC of Kentucky bluegrass with choline chloride and salt stress treatments. **(A)** The content of PG content. **(B)** The content of PI content. **(C)** The content of PS content. **(D)** The content of LysoPC content. All data are means ± SE for three biological replicates. Different letter indicated the significance at P < 0.05.

The different lipid specific molecular classes are shown in **Figures 6**, **7** in Kentucky bluegrass leaves treated with choline chloride and salt stress.

For glycolipid species, the 36:6 lipid species had the highest abundant in all treatments in MGDG and DGDG (**Figure 6A, B**). The content of 34:3 and 36:6 molecular classes in DGDG were decreased and the content of the 36:5 molecular class in MGDG was increased, with or without choline chloride treatment under salt stress condition. The DGDG (34:3, 36:6) content was significantly higher in choline chloride-treated plants under non stress conditions, whereas the DGDG (34:3, 36:6, 36:5) content was significantly higher in choline chloride -treated plants under salt and non-stress conditions. Compared to untreated plants, the MGDG (36:6, 36:5) content was significantly increased in choline chloride -treated plants in under salt-stress. For phospholipid species, under non stress conditions, exogenous choline chloride significantly decreased the PA (34:3, 36:6) content, whereas PA species (34:3, 34:2, 36:5, 36:4, and 36:2) content were lower in choline chloride-treated plants compared nontreated plants under salt stress (**Figure 6C**). The PC (34:3, 34:2, 36:5, 36:4, 36:3, and 36:2) content decreased significantly after salt stress treatment. Under salt stress, the content of PC (36:5 and 36:4) in choline chloride treated plant were significantly higher that of non-treated plants (**Figure 6D**). The PE (34:2, 36:4) content was significantly lower under salt stress than that under nonstress conditions, and PE (34:3, 36:4) was higher in choline chloride -treated plants (**Figure 6F**). For other phospholipid species, a significant decrease in PG (34:4, 34:3, 34:2) and PS (40:3, 40:2) was observed after salt stress and choline chloride treatment significantly increased the PG (34:4, 34:3), PI (34:3, 34:2), and PS (40:3, 40:2) contents under non stress conditions (**Figure 7A-C**). Under salt stress, PI (34:2) and PS (40:3, 40:2) contents were both greatly increased by choline chloride treatment. For lysophospholipids, the content of LysoPC (18:3, 18:2) significantly increased in choline chloride -treated plants under non stress conditions, whereas that of LysoPC (16:0, 18:3, 18:2) significantly increased in choline chloride -treated plants under salt and non-stress conditions (**Figure 7D**). The DGDG, PC, and PE unsaturation were significantly increased, whereas the PG unsaturation was significantly decreased with or without choline chloride treatment after salt stress treatment (**Table S2**). In choline chloride -treated plants, a significantly lower unsaturation (3.02%) was observed in PC under salt stress.

### 3.4 The leaf GB and choline content determination

**Figure 8:**
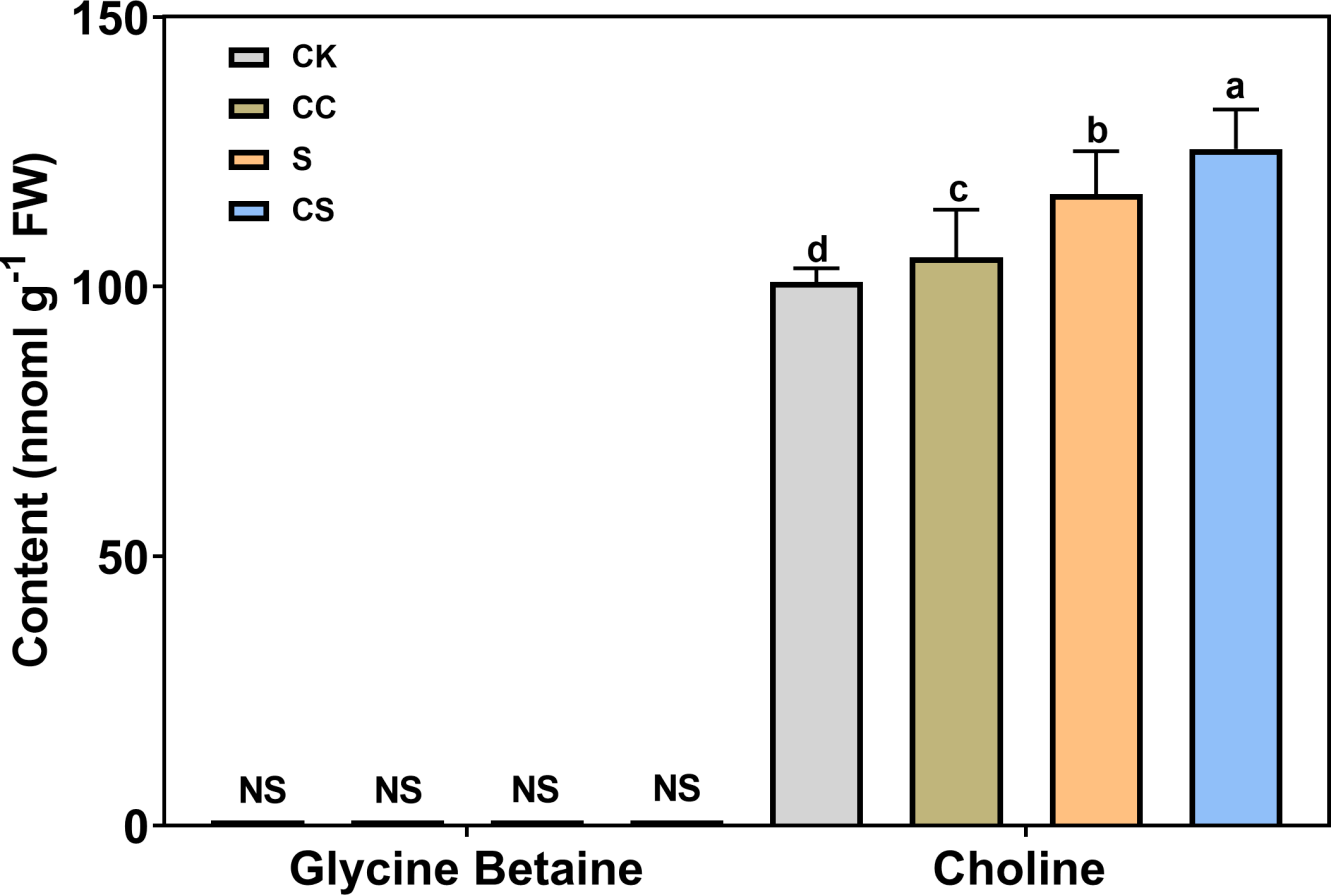
Leaf endogenous GB and choline content with choline chloride and salt stress treatments. NS means undetectable. All data are means ± SE for three biological replicates. Different letter indicated the significance at P < 0.05.

To determine the major pathways regulated by choline chloride, GB and choline contents were analyzed under different treatments (**Figure 8**). The results showed that the GB content were not detected following either salt or choline chloride treatment. Salt stress significantly increased the choline content with or without choline chloride treatment. Exogenous choline chloride significantly increased choline content (4.52% and 7.13%) under nonstress and salt-stress conditions, respectively. These results revealed that exogenous choline chloride could affect endogenous choline content rather than the GB synthesis pathway under stress and nonstress conditions in Kentucky bluegrass.

### 3.5 The expression of GB synthesis and CPD-choline pathway genes

**Figure 9:**
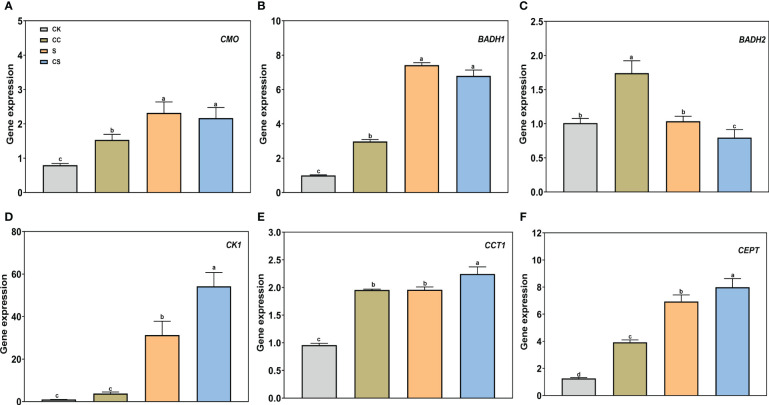
The gene expression of GB and CDP-choline metabolism pathway with choline chloride and salt stress treatments. **(A)** CMO. **(B)** BADH1. **(C)** BADH2. **(D)** CK1. **(E)** CCT1. **(F)** CEPT. All data are means ± SE for three biological replicates. Different letter indicated the significance at P < 0.05.

Furthermore, expression analysis of genes controlling lipid synthesis was performed to determine the molecular mechanisms affected by choline. For genes in the GB synthesis pathway, salt stress greatly increased the expression of *CMO* and *BADH1* and decreased the expression of *BADH2* (**Figures 9A-C**). Exogenous choline chloride upregulated the *CMO* (1.53-fold), *BADH1* (2.97-fold), and *BADH2* (1.74-fold) gene expressions under nonstress conditions. The expression of *CMO* and *BADH1* had no significant changes to exogenous choline chloride after salt stress treatment, whereas *BADH2* gene expression was downregulated by the choline chloride treatment under salt stress. For genes in the CPD-choline pathway, salt stress significantly induced the expression of *Choline kinase* (*CK1*), *CTP-phosphocholine cytidylyltransferase* (*CCT1*), and *Choline/ethanolaminephosphotransferase* (*CEPT*) (**Figures 9D-F**). Under salt stress, the expression of *CK1* was significantly upregulated (73.19%) by choline chloride treatment. The expression of *CCT1* and *CEPT* gene were significantly higher in choline chloride treated plants under nonstress and salt-stress conditions by 104.24% and 209.87% and 14.64% and 15.21%, respectively. These results revealed that choline chloride regulates salt stress in Kentucky bluegrass *via* choline metabolic pathways and not through the GB synthesis pathway.

## 4 Discussion

Soil salinity is an important factor that affecting turfgrass growth and utilization. Kentucky bluegrass is sensitive to salt stress, and mitigation of the detrimental effects of salt stress and improvement in its tolerance is urgently required. Choline has been identified play role in plant salt stress tolerance (Incharoensakdi and Karnchanatat, 2003). In the present study, Fv/Fm, relative water content, chlorophyll content, and OA were increased, and EL were decreased in choline chloride -treated plants in salt treated plants. This illustrated that exogenous choline chloride could improve the salt tolerance of Kentucky bluegrass. Membrane remodeling is an important metabolic regulatory mechanism in plants exposed to salt stress (Guo et al., 2022). In soybeans, a short-term salt stress treatment could trigger a dynamic reprogramming in both galactolipids and phospholipids (Liu et al., 2021). Similar results have been reported in potato (*Solanum tuberosum*) (Yu et al., 2019), cotton (*Gossypium herbaceum*) (Liu et al., 2022), and *Carex rigescens* (Hu et al., 2021). As a precursor, choline is participated in two key pathways including GB metabolism pathway and lipid metabolism pathway in plant (Annunziata et al., 2019). In our study, endogenous GB metabolism pathway had not obviously response to choline application, while lipid (galactolipids and phospholipids) remodeling, were occurred with choline treatment, especially under salt stress. These results show that choline-mediated lipid metabolism is the central salt tolerance mechanism in Kentucky bluegrass.

Our data showed that the glycolipid and phospholipid contents were increased with choline chloride treatment. Choline alters the reorganization of lipid composition to protect membrane from the injury caused by salt stress, and similar results have been reported for the seashore paspalum (Gao et al., 2020). Plant glycolipids contribute to the formation of stacked thylakoid membranes and DGDG and MGDG content have been reported to decrease after salt stress in rice (Wang et al., 2014) and *Suaeda salsa* (Sui et al., 2010). In our study, an increase in the content of DGDG and MGDG was observed under salt stress with choline chloride treatment. DGDG and MDGD are likely to act as stabilizers in the organization of thylakoid membranes and are indispensable components of the light-harvesting complex II in plant photosynthesis (Guo et al., 2019). Furthermore, plants can alter the thylakoid membrane lipids component to resist abiotic stress. The content of MGDG 36:6, 36:5, and DGDG 34:3, 36:6, and 36:5 was significantly higher in choline chloride treated plants under salt stress, suggesting that these glycolipid species make a difference in choline mediated salt tolerance improvement. Similarly, the levels of 18:2-containing glycolipids (like MGDG-36:5 and DGDG-36:5) were upregulated by different abiotic stresses in tall fescue (*Festuca arundinacea*) (Zhang et al., 2019), *C. rigescens* (Hu et al., 2021), and rice (Wang et al., 2019). These results suggest that unsaturated 18:2-containing glycolipids might be significant in abiotic stress responses in plants.

Under salt stress condition, the PC content was significantly higher in choline chloride treated plants. PC participate in abiotic stress tolerance in plants (Tasseva et al., 2004). Studies have confirmed membrane PC has positive role in plant salt tolerance in *Arabidopsis thaliana* (Qiao et al., 2018), cordgrass (*Spartina patens*) (Wu et al., 2005) and tomato (*Lycopersicon esculentum*) (Kerkeb et al., 2001). In addition, in *Catharanthus roseus* and *Mesembryanthemum crystallinum* which had high salt tolerence (Elkahoui et al., 2004; Barkla et al., 2018), PC species (36:5 and 36:4) increased after salt stress compared to those in salt-sensitive plants, suggesting that they are involved in salt stress adaptation (Magdy et al., 1994). In the present study, the PC unsaturation index decreased with exogenous choline chloride. In PC-specific molecular species, the abundance of 16:0 acyl chains were higher than that of 18:3/18:2 acyl chains after choline chloride treatment. High lipid saturation contributes to plant cell membrane stability and integrity under abiotic stress (Li et al., 2020), indicating that these saturated lipid species may play an important role in choline-mediated salt stress tolerance in Kentucky bluegrass.

PE and PC are the major phospholipid components of extraplastidial membranes, and PE can regulate stress signal transduction under hyperosmotic stress *via* phospholipase C (Pokotylo et al., 2014; Singh et al., 2015). Under salt stress, the PE content was significantly increased in choline chloride treated plants. Studies have reported that PE increases in association with PC, resulting in abiotic stress due to increased membrane fluidity (Higashi and Saito, 2019). The choline group of PC is a bilayer-forming lipid, whereas the ethanolamine group of PE is a non-lamellar-forming lipid, which affects the formation of the bilayer phase and preserves membrane fluidity (Toumi et al., 2008; Narayanan et al., 2018). Therefore, the PC/PE ratio may reflect a cellular response to maintain membrane stability and balance between the membrane and storage lipids (Narayanan et al., 2018). In the present study, the PC/PE ratio increased in response to salt stress with exogenous choline chloride, illustrating that the elevated abundance of PC rather than PE with choline chloride treatment could help maintain the membrane integrity and fluidity in Kentucky bluegrass.

PA has been reported to crucial for salt stress responses by helping maintain ion homeostasis (Yao and Xue, 2018; Liu et al., 2019). In addition, PA is a major intermediate that guiding the PC and PE biosynthesis from the CDP-choline and CDP-ethanolamine pathways, respectively (Carman and Han, 2009). The PA content and specific molecular species decreased under salt stress in Kentucky bluegrass, and the result was the opposite of the PC and PE variation. Under abiotic stress conditions, phospholipase D could hydrolyze of PE and PC and produce PA in plants (Jiang et al., 2019). Therefore, these results suggest that choline can improve salt tolerance in Kentucky bluegrass by promoting PA downstream of PC and PE synthesis through the CDP-choline pathway.

The phosphatidylserine (PS) content can be regulated by abiotic stress in plants (Higashi and Saito, 2019). PS includes a large amount of lipid species, such as PS (40:3), which contains very-long-chain fatty acids and has been reported to increase in heat stress-susceptible wheat genotype plants (Narayanan et al., 2016). In the present study, choline chloride treatment increased the PS (40:3 and 40:2) content under salt stress conditions. However, the function of the increased PS content under salt stress is unknown. A previous study reported that PS decarboxylase (PSD) converts PS to PE through endoplasmic reticulum (Yamaoka et al., 2011). Therefore, the changes in PS in plants exposed to salinity stress with choline chloride treatment may rely on the PSD-mediated metabolic pathway and, together with PE, may play a role in membrane stability.

LysoPC is a lipid class with only one fatty acyl chain on the glycerol moiety of glycerolipids, which is surface-active, alters membrane fluidity, and affects membrane receptor functions (Zhang et al., 2018). In the present study, choline chloride treatment increased LysoPC content under salt stress conditions. In *Schizochytrium* sp., LysoPC lipid species were significantly enriched in the choline metabolism pathway, contributing to improved salt stress tolerance (Jiang et al., 2019). However, the role of LysoPC in stress tolerance in plants remains unclear. Plant Ca^2+^ plays a significant role in cell membrane integrity, and endogenous Ca^2+^ levels may decrease during salt stress (Hashemipetroudi et al., 2022). A previous study found that the content of LysoPC increased in response to Ca^2+^ deprivation (IsHizuKA et al., 2000), demonstrating that LysoPC might play an essential role in Ca^2+^ maintaining plasma membrane stability.

In summary, choline chloride increased the salt tolerance of Kentucky bluegrass by increasing Fv/Fm, relative water content, chlorophyll content, and OA and reducing leaf EL. Lipidomic results showed that the content of total MGDG, DGDG, PC, PE, PS, and LysoPC were increased, and PA content was decreased with exogenous choline chloride when exposed to salt stress. The leaf GB content and synthesis related genes had no response to exogenous choline chloride, indicating that choline triggered lipid metabolism was a primary mechanism in Kentucky bluegrass response to salt stress condition The specific lipid molecular species of glycolipids (MGDG 36:6, 36:5, and DGDG 34:3, 36:6, 36:5), phospholipids (PC 36:5, 36:4, PE 34:3, 34:2, 36:5, 36:4, PI 34:2, and PS 40:3, 40:2), and lysophospholipids (LysoPC 16:0, 18:3, 18:2) were increased exposed to exogenous choline chloride. Our results provide lipid biomarkers for further improvement of salt-sensitive grass species.

## Data availability statement

The raw data supporting the conclusions of this article will be made available by the authors, without undue reservation.

## Author contributions

ZZ: Writing – original draft, Writing – review and editing, Software, Data curation, Formal Analysis, Validation. YL: Validation, Writing – review and editing, Data curation, Formal Analysis, Software. XM: Data curation, Software, Writing – review and editing, Formal Analysis. YL: Writing – review and editing, Investigation, Validation. CZ: Validation, Writing – review and editing, Data curation. GY: Writing – review and editing, Resources, Supervision. ZW: Supervision, Writing – review and editing, Funding acquisition. KZ: Funding acquisition, Supervision, Writing – review and editing, Software, Writing – original draft.
