# Supplementary material for: Physiological and lipidomic response of exogenous choline chloride alleviating salt stress injury in Kentucky bluegrass (Poa pratensis)
Source: Front Plant Sci. 2023 Aug 31;14:1269286. doi: 10.3389/fpls.2023.1269286 (PMC10501137; doi:10.3389/fpls.2023.1269286)
Supplement: Supplementary file 1 [file Table_1.docx]

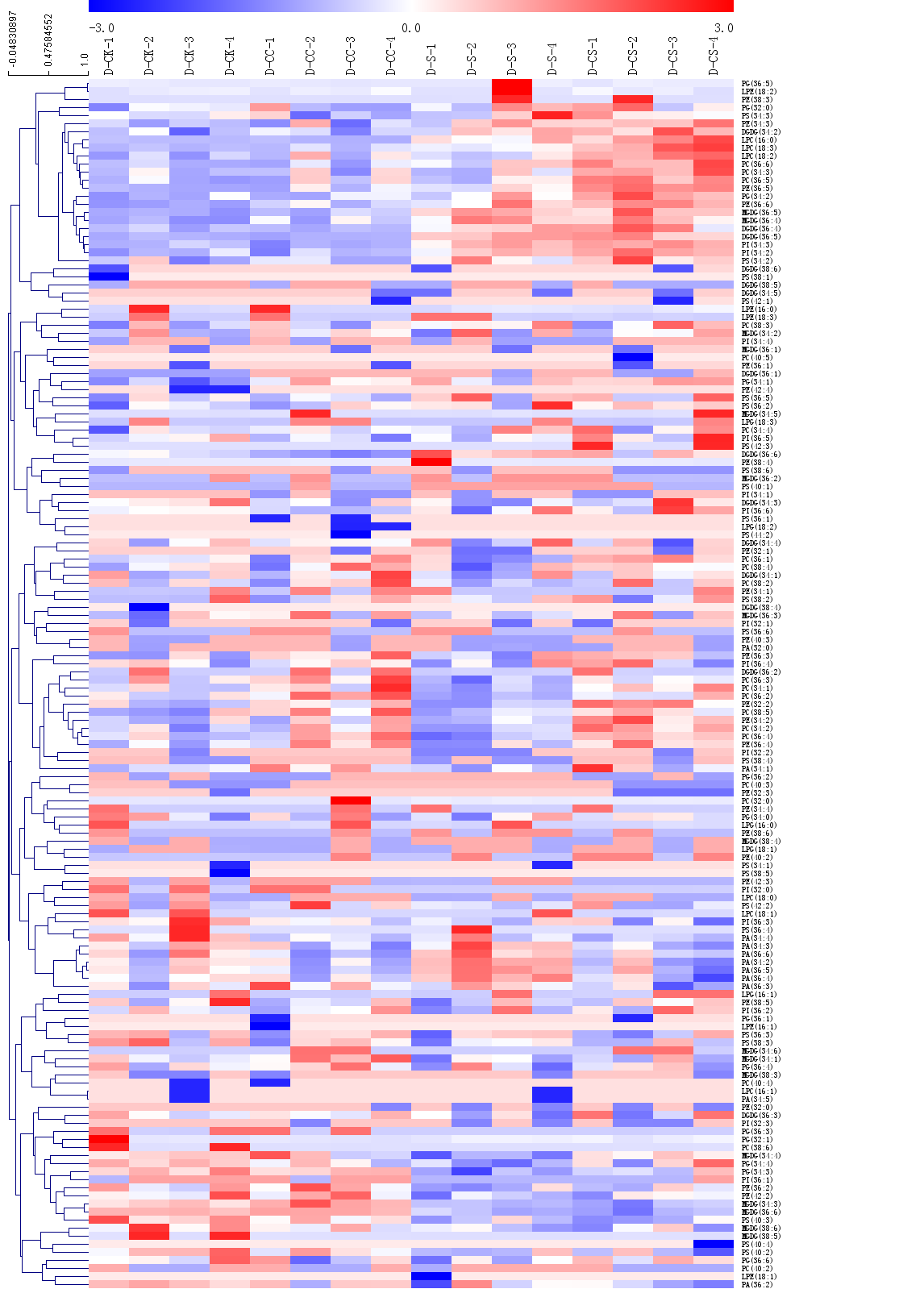


Fig. S1 Overview of all the lipids detected in Kentucky bluegrass leaves in response to choline application and salt stress

Note: CK, control optimal condition; CC, choline treatment; S, salt stress; CS, choline treatment + salt stress. MGDG, monogalactosyl diacylglycerol; DGDG, digalactosyl diacylglycerol; PA, phosphatidic acid; PC, phosphatidylcholine; PE, phosphatidylethanolamine; PI, phosphatidylinositol; PG, phosphatidylglycerol; PS, phosphatidylserine; LPC, Lysophosphatidylcholine,; LPE, Lysophosphatidylethanolamine; LPG, Lysophosphatidylglycerol. Data were calculated by z-score normalization.
