# Supplementary material for: Physiological and lipidomic response of exogenous choline chloride alleviating salt stress injury in Kentucky bluegrass (Poa pratensis)
Source: Front Plant Sci. 2023 Aug 31;14:1269286. doi: 10.3389/fpls.2023.1269286 (PMC10501137; doi:10.3389/fpls.2023.1269286)
Supplement: Supplementary file 3 [file Table_3.docx]

| **Lipid class** | **Treatments** | | | |
| --- | --- | --- | --- | --- |
|  | **CK** | **CC** | **S** | **CS** |
| Glycolipids |  |  |  |  |
| DGDG | 5.10 ± 0.01 b | 5.14 ± 0.01 b | 5.12 ± 0.02 b | 5.19 ± 0.01 a |
| MGDG | 5.76 ± 0.01 a | 5.78 ± 0.00 a | 5.69 ± 0.01 b | 5.70 ± 0.01 b |
| Phospholipids |  |  |  |  |
| PA | 3.26 ± 0.01 ab | 3.21 ± 0.01 b | 3.27 ± 0.01 a | 3.25 ± 0.02 ab |
| PC | 3.23 ± 0.02 c | 3.21 ± 0.01 c | 3.41 ± 0.01 a | 3.31 ± 0.00 b |
| PE | 3.24 ± 0.02 b | 3.28 ± 0.01 b | 3.35 ± 0.02 a | 3.36 ± 0.01 a |
| PI | 2.40 ± 0.01 a | 2.43 ± 0.01 a | 2.41 ± 0.01 a | 2.42 ± 0.01 a |
| PG | 2.98 ± 0.01 a | 3.04 ± 0.02 a | 2.91 ± 0.03 b | 2.91 ± 0.02 b |
| PS | 1.85 ± 0.05 a | 1.86 ± 0.01a | 1.83 ± 0.04 a | 1.87 ± 0.03 a |

**Table S2 The unsaturation index level of lipid classes** **in response to salt tress and exogenous choline**

CK, control optimal condition; CC, choline treatment; S, salt stress; CS, choline treatment+ salt stress. DGDG, digalactosyl diacylglycerol; MGDG, monogalactosyl diacylglycerol; PA, phosphatidic acid; PC, phosphatidylcholine; PE, phosphatidylethanolamine; PI, phosphatidylinositol; PG, phosphatidylglycerol; PS, phosphatidylserine. All data are means ± SE for three biological replicates. Different letter indicated the significance at *P* < 0.05.
